# Supplementary material for: Association between oral microbiome and seven types of cancers in East Asian population: a two-sample Mendelian randomization analysis
Source: Front Mol Biosci. 2023 Nov 21;10:1327893. doi: 10.3389/fmolb.2023.1327893 (PMC10702768; doi:10.3389/fmolb.2023.1327893)
Supplement: Supplementary file 1 [file DataSheet1.ZIP › Supplementary Materials/MR plots of saliva/saliva═╝/Breast cancer/pheno.2558_to_breast cancer_leave_one_out.pdf]

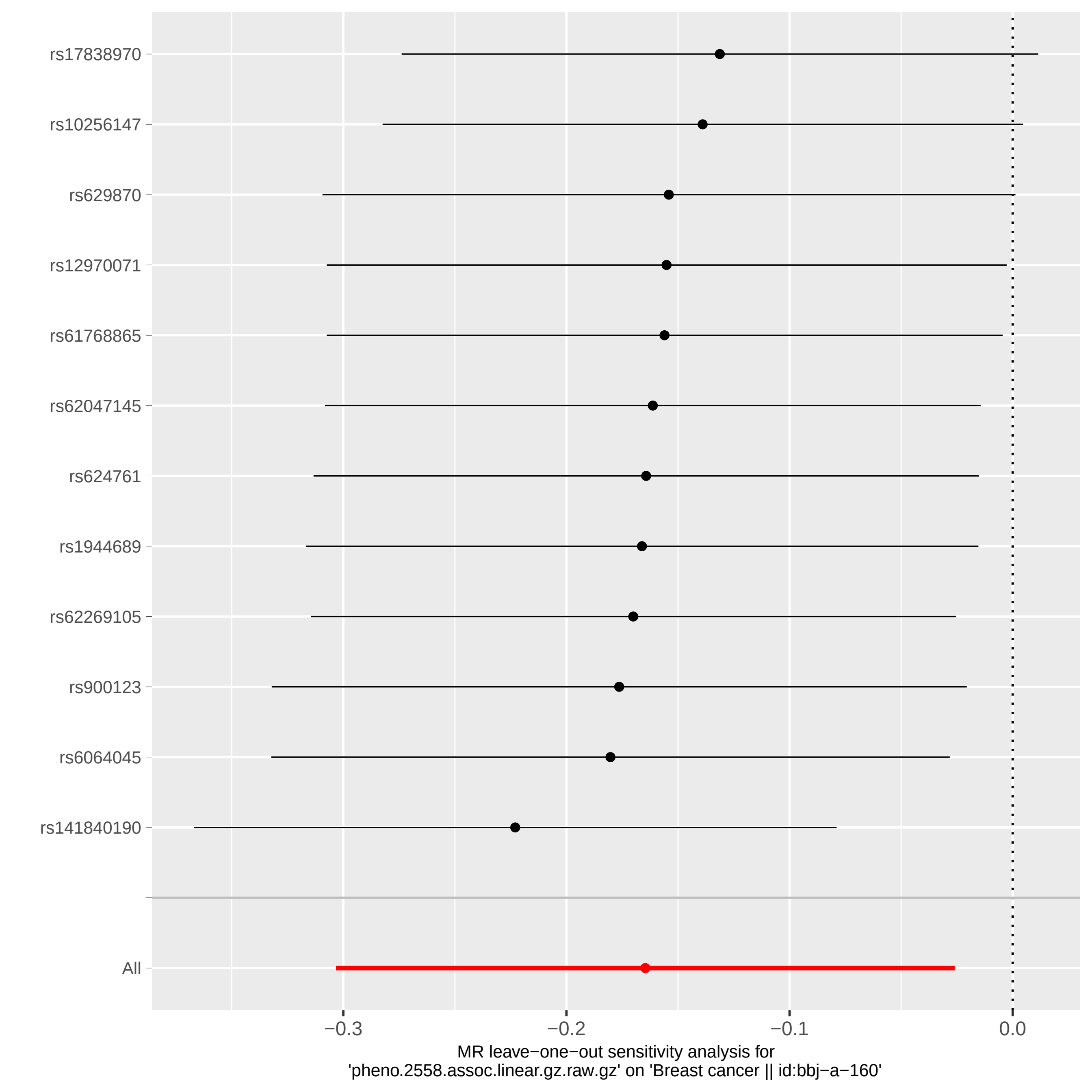
rs17838970
rs10256147
rs629870
rs12970071
rs61768865
rs62047145
rs624761
rs1944689
rs62269105
rs900123
rs6064045
rs141840190
All
−0.3
−0.2
−0.1
0.0
MR leave−one−out sensitivity analysis for
'pheno.2558.assoc.linear.gz.raw.gz' on 'Breast cancer || id:bbj−a−160'
